# High-resolution peripheral quantitative computed tomography in children with osteogenesis imperfecta

**DOI:** 10.1007/s00247-020-04736-8

**Published:** 2020-07-01

**Authors:** David J. Fennimore, Maria Digby, Margaret Paggiosi, Paul Arundel, Nick J. Bishop, Paul Dimitri, Amaka C. Offiah

**Affiliations:** 1 Academic Unit of Child Health, University of Sheffield, Damer Street, Sheffield, S10 2TH UK; 2 The Mellanby Centre for Bone Research, Academic Unit of Bone Metabolism, Department of Oncology and Metabolism, The University of Sheffield, Sheffield, UK; 3 Sheffield Children’s NHS Foundation Trust, Western Bank, Sheffield, S10 2TH UK

**Keywords:** Bone mechanical properties, Bone microstructure, Children, Dual-energy X-ray absorptiometry, Feasibility study, High-resolution peripheral quantitative computed tomography, Osteogenesis imperfecta

## Abstract

Bone health in children with osteogenesis imperfecta is monitored using radiographs and dual-energy X-ray absorptiometry, which have limitations. High-resolution peripheral quantitative CT can non-invasively derive bone microarchitectural data. Children with severe osteogenesis imperfecta have fragile deformed bones, and positioning for this scan can be difficult. We assessed the feasibility of high-resolution peripheral quantitative CT in nine children aged 9–15 years with osteogenesis imperfecta and compared results with dual-energy X-ray absorptiometry and with healthy controls. All nine recruited children were successfully scanned and showed no preference for either modality. It therefore appears feasible to perform high-resolution peripheral quantitative CT in children with osteogenesis imperfecta aged 9 years and older. Future studies should focus on understanding the clinical implications of the technology in this patient cohort.

## Description

Bone quality encompasses all material and geometric factors contributing to fracture resistance [1]. A decrease in bone strength results in greater risk of fracture. Osteogenesis imperfecta is characterised by defective Type I collagen and fragile bones that frequently fracture. There are various types of osteogenesis imperfecta, ranging in severity from mild to perinatally lethal. Routine monitoring of these children varies among centres but usually includes 6-monthly dual-energy X-ray absorptiometry (for bone mineral density assessment) and either annual lateral spine radiographs or 6-monthly lateral dual-energy X-ray absorptiometry of the spine (for detecting vertebral fractures). Given its limitations in assessing bone density, conventional radiography is only performed when fracture or other pathology (e.g., basilar invagination) is suspected.

The International Society for Clinical Densitometry states that radiologic investigations should be used to identify children who might benefit from clinical intervention [2]. Dual-energy X-ray absorptiometry is a low-cost technique that exposes children to low radiation dose. However, it can only produce 2-D information, is dependent upon patient size, does not provide separate cortical and trabecular information, does not produce information regarding bone microstructure and does not predict fracture risk [3].

The primary aim of this study was to assess the feasibility of performing high-resolution peripheral quantitative CT scans in children with osteogenesis imperfecta, and to compare results with values in a cohort of age- and sex-matched healthy children.

High-resolution peripheral quantitative CT (Xtreme CT; SCANCO Medical AG, Brüttisellen, Switzerland) produces images with an isotropic voxel size of 82 μm^3^ (XtremeCT) or 64 μm^3^ (XtremeCT II) at low radiation dose (3 microSv per scan). Cortical and trabecular parameters of ultra-distal radii and tibiae are measured, and other bone microarchitectural measures are derived using specific algorithms. Although it cannot replace histomorphometry, it may be a non-invasive alternative [3], but currently in paediatrics it is predominantly (if not exclusively) used as a research tool. Studies have shown that high-resolution peripheral quantitative CT can demonstrate bone microarchitecture and assess bone strength in healthy children [4].

Patients aged 7–15 years attending the osteogenesis imperfecta clinic at Sheffield Children’s Hospital were eligible for the study. A total of 48 patients were approached. We recorded each child’s osteogenesis imperfecta type, ethnicity, drug history, limb dominance, height and weight.

Exclusion criteria included being unwilling or unable to provide informed consent, previous enrolment into the study and inability to travel to the scanner with minimal risk. Intramedullary rods were also an exclusion criterion because of the negative effect of beam artefact that they cause. If children fractured one limb in the 6 weeks prior to the scan or had a plaster cast applied to one limb, then only that limb was excluded.

Briefly, as previously described by Paggiosi et al. [5] and Walsh et al. [6], each child had high-resolution peripheral quantitative CT scans of both ultra-distal forearms and ultra-distal ankles using the Xtreme CT I (SCANCO Medical) at the Northern General Hospital, Sheffield. Scans were performed and quality was assessed and analysed by an experienced, trained technician. The limb to be scanned was placed in a carbon fibre cast and then into the gantry of the scanner. A scout view scan was obtained prior to the main scan to establish the starting location for the scan: 1 mm proximal from the proximal point of the distal epiphyseal growth plate [6, 7]. A 9.8-mm section of the ultra-distal radius or tibia was then scanned in a proximal direction. Grading was performed as per Engelke et al. [8] with grades representing the level of movement artefact: none (Grade 1), slight (Grade 2), moderate (Grade 3) or unacceptable (Grade 4). Grades 1, 2 and 3 were accepted; however, those graded 4 were not used and such scans were repeated once (a maximum of two measurements were performed at each anatomical site). Refer to Paggiosi et al. [5] for example images of each grade.

We used standard SCANCO Medical software to perform image segmentation and analysis, producing total trabecular and cortical volumetric bone mineral density (mgHA/cm^3^), cortical thickness (mm) and perimeter (mm), and total trabecular and cortical area (mm^2^). Additionally, we recorded microarchitectural parameters including trabecular thickness (mm), number (1/mm), separation (mm), inhomogeneity (mm) and bone volume fraction. Extended cortical analysis was completed using specialist SCANCO Medical software and the approach described by Burghardt et al. [9] to quantify cortical porosity. Application of microfinite element analysis produced values describing the stiffness (kN/mm) and ultimate failure load (kN) of each bone. The ratio of load taken by trabeculae in relation to total proximal or distal load (%) was determined. Von Mises stresses (MPa) were also determined to ascertain the load at which a combination of stresses in different directions at any one point in the bone (radius or tibia) would cause failure of that bone.

Dual-energy X-ray absorptiometry parameters including bone mineral content less head (g), total bone mineral density less head (g/cm^2^), total bone area less head (cm^2^) and z-scores were extracted from the patients’ most recent routine scan for comparison between groups. All z-scores were age- and body-size-corrected. The scanner was a Lunar device (GE Healthcare, Waukesha, WI). All scans were performed within 6 months of the high-resolution peripheral quantitative CT scans.

Each patient received a non-validated questionnaire that asked them about their experience of being scanned, to rate their experience of the high-resolution peripheral quantitative CT and dual-energy X-ray absorptiometry scans and to record how comfortable they found remaining motionless for each scan. Three options were given for each of the four questions above as follows: “Very uncomfortable”, “Uncomfortable” and “No problem”. Finally, the children were asked which test they preferred, or if they had “no preference”, and why.

Control data were obtained from a previous study of healthy children [10]. Controls were matched to patients by age, sex and pubertal stage. No controls had received bisphosphonate treatment. Dual-energy X-ray absorptiometry scans of controls were performed using a Discovery A scanner (Hologic, Bedford, MA); therefore, a conversion factor was applied to allow for comparison with variables derived from the GE device [11].

Leeds West Research Ethics Committee and UK Health Research Authority granted approval for this study, which we registered with the local Research and Development Department. We obtained full informed written consent and assent prior to performing any of the study procedures. This research project was conducted in accordance with the standards set out during the International Conference on Harmonisation and using good clinical practice.

Statistical analyses were not performed as part of this feasibility study. However, for the purposes of data presentation, we grouped children by osteogenesis imperfecta type (I and IV = mild, III and V = severe) and by whether they had ever received bisphosphonate therapy.

Of the 48 children with osteogenesis imperfecta approached between December 2014 and April 2015, nine were recruited, of whom five (56%) were male and all were Caucasian. The children ranged in age from 9 years to 15 years, with a median of 13 years. Each child had a number of previous fractures ranging from 2 to 25. Both children with severe osteogenesis imperfecta and five of the seven (71%) with mild disease were on or had received bisphosphonate treatment. Table 1 summarises the characteristics of patients and healthy controls [10].

**Table 1:** Median values [interquartile range] for characteristics of children with osteogenesis imperfecta and controls

Characteristic	Total children with osteogenesis imperfecta (*n*=9)	Mild osteogenesis imperfecta (*n*=7)	Severe osteogenesis imperfecta (*n*=2)	Controls^a^ (*n*=9)
Age (years)	10.7 [9.5 to 12.5]	11.5 [9.2 to 15.2]	10.6 [10.6 to 10.7]	10.6 [10.0 to 12.4]
Height (cm)	130 [130 to 150]	130 [130 to 160]	120 [110 to 130]	150 [140 to 150]
Height z-score	−1.220 [−1.542 to −0.779]	−1.001 [−1.542 to −0.063]	−3.097 [−4.969 to −1.225]	0.525 [−0.069 to 0.842]
Weight (kg)	32.1 [30.3 to 36.3]	32.1 [30.3 to 61.0]	29.4 [26.0 to 32.8]	36.6 [33.2 to 41.9]
Weight z-score	−0.305 [−1.070 to 0.602]	0.444 [−1.070 to 1.049]	−0.988 [−1.671 to −0.305]	0.366 [−0.163 to 1.252]
Body mass index (kg/m^2^)	18.3 [17.2 to 22.0]	17.5 [16.8 to 23.8]	20.2 [18.3 to 22.1]	16.1 [15.9 to 20.1]
Body mass index z-score	0.745 [−0.171 to 1.649]	0.745 [−0.297 to 1.819]	1.043 [0.437 to 1.649]	−0.067 [−0.795 to 1.209]
Bisphosphonate treatment duration (years)	5.7 [1.7 to 9.4]	2.9 [0.9 to 8.1]	10.5 [0 to 0]	0 [0 to 0]
Previous fractures (*n*)	10 [8 to 18]	9 [2 to 18]	20.5 [16 to 25]	0 [0 to 0]

^a^Control data from previous study [10]

**Fig. 1:**
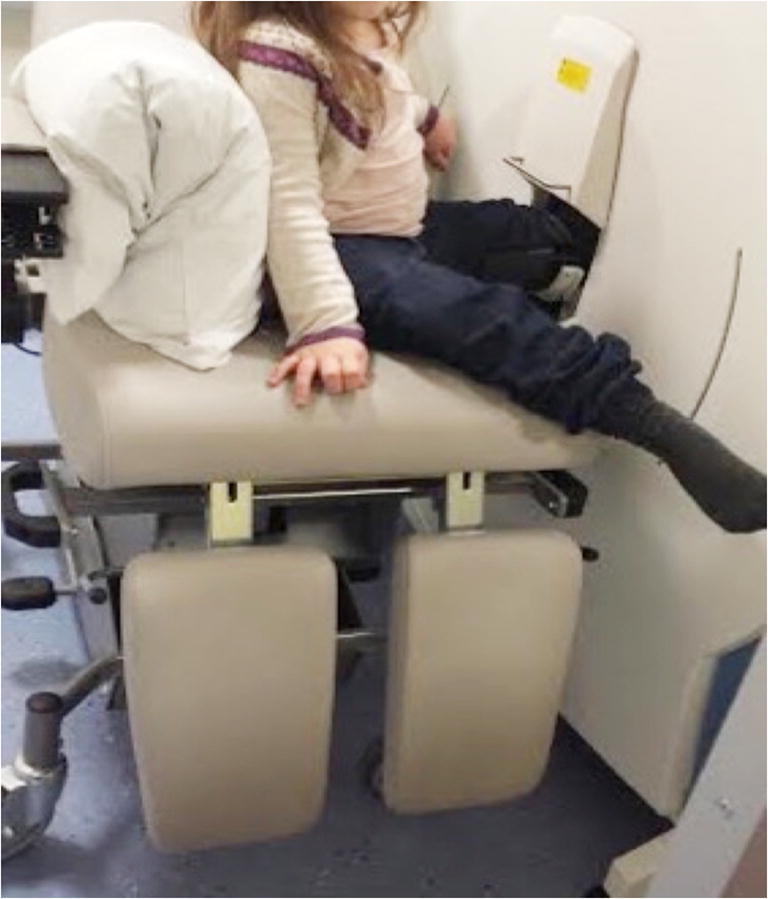
Clinical photograph of a 9-year-old girl with osteogenesis imperfecta. The chair had to be turned by 90° and extra pillows placed along the arm rest to adequately position her for scanning of her distal tibia

**Fig. 2:**
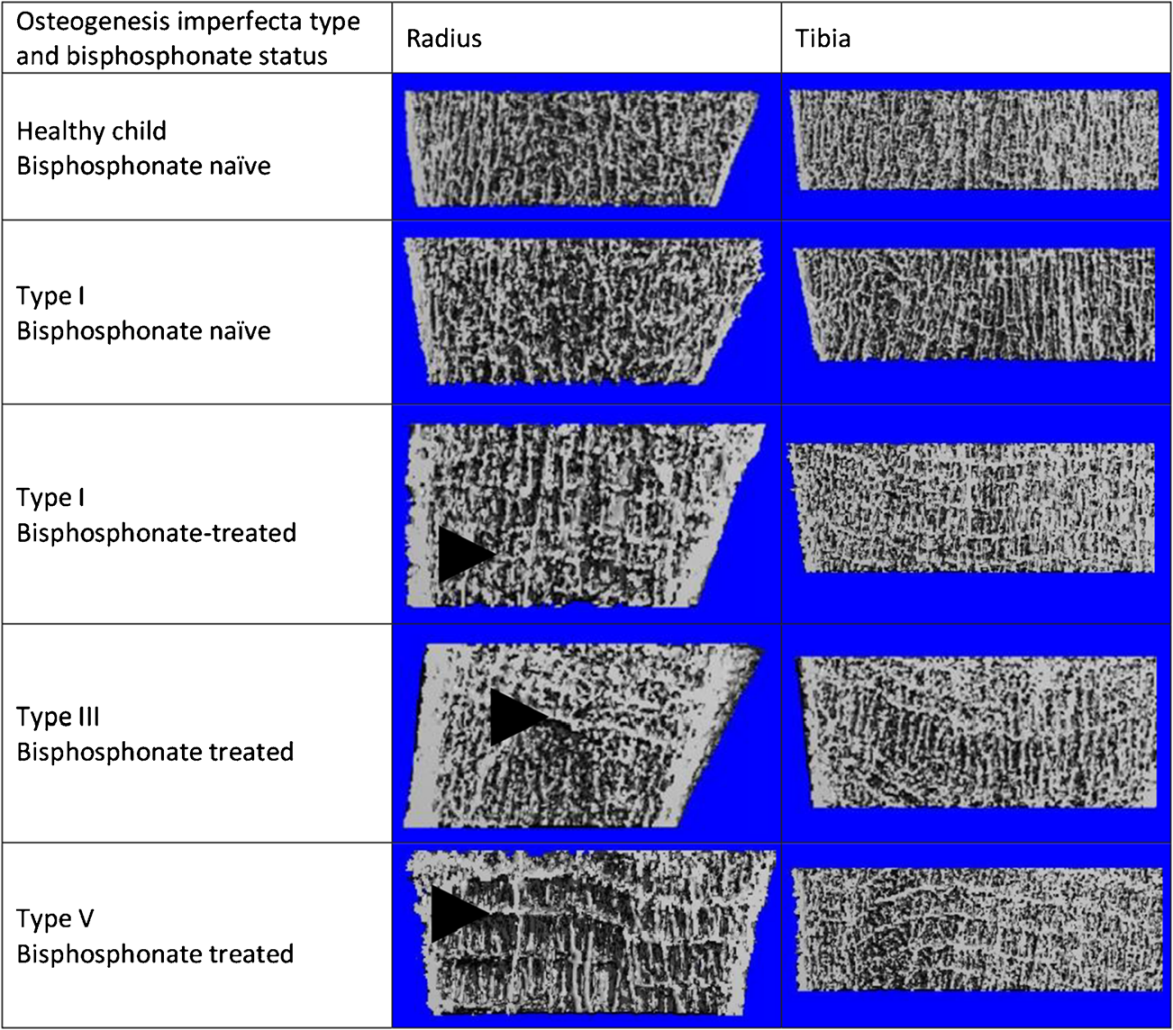
Coronal slices of non-dominant radii and non-dominant tibiae produced by high-resolution peripheral quantitative CT scans of children with osteogenesis imperfecta. Note the high resolution of the images and the horizontal bisphosphonate, or “zebra”, lines in bisphosphonate-treated children (*arrowheads*), which should not be mistaken for trabeculae. The bisphosphonate lines appear more pronounced in the radius, possibly because of image magnification, although increased metabolic activity at the wrist compared to the ankle might play a role

Of the nine children, seven had all four limbs successfully scanned. One of the remaining two children had three limbs scanned because their non-dominant ankle was encased in plaster. The other had metal plates in both ankles so only had both radii imaged. A total of 33 scans were performed. At the first attempt, 26 were deemed to be of adequate quality (Grades 1, 2 or 3), but 7 repeat scans were required because of significant movement artefact (Grade 4). Six of the seven repeated scans were of the radii. The mean scan time was 2.8 min. Because the scanner was designed for adults, there were complexities positioning the children in the chair at the correct height for their limb to enter the gantry of the scanner, and various modifications were required (Fig. 1). Figure 2 shows high-resolution peripheral quantitative CT images of radii and tibiae in children affected by different types of osteogenesis imperfecta.

The questionnaire results showed no significant difference in preference for either scan. One of the nine children found dual-energy X-ray absorptiometry “uncomfortable”, and when questioned about remaining motionless for this scan, three children found it was “uncomfortable” and six “no problem”. Regarding high-resolution peripheral quantitative CT, all nine children indicated “no problem” with the scanning, and when questioned about remaining motionless, two said it was “uncomfortable” and seven “no problem”.

## Discussion

The principal aim of this study was to investigate the feasibility of high-resolution peripheral quantitative CT imaging for the assessment of bone microarchitecture and strength in children with osteogenesis imperfecta, especially regarding achievement of adequate positioning. Even the two severely affected children were scanned using the standard Xtreme CT chair with adequate quality scans achieved. Good-quality scans were achieved in all limbs scanned on either the first (79%) or second (21%) attempt. The shortest child (108 cm) required additional pillows because of short stature; however, all would have benefitted from a seatbelt to aid patient stability and safety in the chair when it was raised to the level of the scanner gantry.

Prior to scanning we anticipated that keeping the children sufficiently still for obtaining diagnostic-quality images was going to be difficult, but the addition of sponges inside the carbon-fibre casts aided children to be comfortable and still during the scan. Both the scanner and the casts were made for adult use, so child-size equipment would also improve the scanning of these children. Achieving adequate positioning was expected to be complex because of the children’s deformities from previous fractures; however, none of the deformities was severe enough to prevent adequate positioning and entry into the gantry of the scanner. One Type V patient was unable to fully extend the elbows because of bilateral radial head dislocation; however, this caused no issues when positioning the child for the scan. No children were harmed by the scanning procedure.

Scanning all four limbs of children with osteogenesis imperfecta had not been performed before, to the best of the authors’ knowledge, and it allowed the authors to assess for differences between sites and sides in the same child. There were no differences in left/right results (as expected, because none had fractures at the imaged sites within 6 weeks of recruitment and interval growth would have allowed the sites of any previous fracture to have extended beyond the ultra-distal portion that was imaged of wrists/ankles).

Individual trabeculae within a given bone are sometimes of differing thickness and microarchitectural property. The extent to which the trabeculae differ is termed “trabecular inhomogeneity”. This is important because bones can only be as strong as their weakest trabeculae [12]. Children with osteogenesis imperfecta had greater trabecular inhomogeneity at the dominant distal radius than healthy controls, supporting the notion that trabecular structure plays an important role in bone strength and that affected children still produce inadequate Type I collagen regardless of bisphosphonate treatment. The role of bisphosphonates on trabecular inhomogeneity is not known. No differences were found between bisphosphonate-treated and bisphosphonate-naïve children, supporting the current practice of selecting patients for bisphosphonate therapy based on fracture history rather than osteogenesis imperfecta type.

More children stated that remaining motionless was “no problem” for high-resolution peripheral quantitative CT, possibly because of the use of immobilisation casts and shorter scan time. However, there was no difference regarding patient preference for either modality.

Dual-energy X-ray absorptiometry provides an age- and gender-related assessment of bone mass that does not distinguish trabecular from cortical bone and provides no information about bone microarchitecture (Table 2) [10]. It is influenced by bone size, which is influenced by body size. Contrastingly, high-resolution peripheral quantitative CT can provide age- and body-size-independent assessment of bone mass and also provides information about microarchitecture. Therefore, any condition in which reduced bone mass or altered bone architecture is suspected, including recurrent fractures or risk factors of underlying bone disease, might have their management enhanced by assessment with high-resolution peripheral quantitative CT because it might detect loss of trabecular bone sooner than dual-energy X-ray absorptiometry. However, unless high-resolution peripheral quantitative CT results are shown to predict fracture risk, it will not completely replace dual-energy X-ray absorptiometry because it only provides data localised to the extremities (Tables 3, 4 and 5) [10].

**Table 2:** Dual-energy X-ray absorptiometry values for patients and controls

Group	Total children with osteogenesis imperfecta (*n*=9)	Mild osteogenesis imperfecta (*n*=7)	Severe osteogenesis imperfecta (*n*=2)	Control^a^ (*n*=9)
Total bone mineral content less head (g)	800.4 [674.2 to 869.0]	800.4 [674.2 to 1,294.9]	699.0 [535.2 to 862.8]	974.9 [805.6 to 1,296.1]
Total bone mineral density less head (g/cm^2^)	0.656 [0.646 to 0.699]	0.656 [0.571 to 0.737]	0.657 [0.646 to 0.668]	0.687 [0.594 to 0.782]
Total bone area less head (cm^2^)	1,219.0 [1,128.0 to 1,345.0]	1,219.0 [1,128.0 to 1,737.0]	1,060.0 [829.0 to 1,291.0]	1,175.9 [1,121.9 to 1,356.7]
Z-score (bone mineral density less head)	−1.4 [−1.4 to −1.0]	−1.4 [−1.7 to −0.3]	−1.3 [−1.4 to −1.2]	−0.7 [−1.2 to −0.6]

Data shown as median [interquartile range]

^a^Control data from previous study [10]

**Table 3:** High-resolution peripheral quantitative CT results for the non-dominant ultra-distal radius for patients and controls

Parameter	Osteogenesis imperfecta	Control^a^
Total volumetric bone mineral density (mgHA/cm^3^)	248.9 [232.5 to 264.3]	237.6 [233.4 to 247.8]
Cortical volumetric bone mineral density (mgHA/cm^3^)	688.3 [654.2 to 742.6]	625.4 [610.9 to 719.6]
Trabecular volumetric bone mineral density (mgHA/cm^3^)	153.1 [121.3 to 171.8]	156.6 [143.5 to 170.3]
Cortical thickness (mm)	0.43 [0.35 to 0.67]	0.26 [0.22 to 0.47]
Cortical porosity (no units)	0.046 [0.044 to 0.062]	0.037 [0.030 to 0.049]
Trabecular number (1/mm)	2.07 [1.94 to 2.08]	1.97 [1.92 to 2.25]
Trabecular thickness (mm)	0.061 [0.052 to 0.074]	0.063 [0.058 to 0.066]
Trabecular separation (mm)	0.427 [0.414 to 0.464]	0.440 [0.382 to 0.467]
Trabecular inhomogeneity (mm)	0.191 [0.181 to 0.350]	0.164 [0.153 to 0.176]
Stiffness (kN/mm)	36.76 [32.22 to 50.51]	44.00 [38.41 to 55.46]
Ultimate failure load (kN)	1.88 [1.78 to 2.45]	2.29 [2.03 to 2.85]

Data are shown as median [interquartile range]

^a^Control data from previous study [10]

**Table 4:** High-resolution peripheral quantitative CT results for the non-dominant ultra-distal tibia for children with osteogenesis imperfecta and controls

Parameter	Osteogenesis imperfecta	Control^a^
Total volumetric bone mineral density (mgHA/cm^3^)	607.5 [588.1 to 658.6]	585.8 [546.2 to 634.5]
Cortical volumetric bone mineral density (mgHA/cm^3^)	175.3 [138.2 to 270.0]	194.1 [155.4 to 219.8]
Trabecular volumetric bone mineral density (mgHA/cm^3^)	0.32 [0.22 to 0.44]	0.21 [0.14 to 0.36]
Cortical thickness (mm)	97.6 [96.7 to 105.8]	111.1 [100.5 to 113.0]
Cortical porosity (no units)	0.146 [0.144 to 0.149]	0.145 [0.142 to.153]
Trabecular number (1/mm)	0.064 [0.060 to 0.078]	0.071 [0.058 to 0.075]
Trabecular thickness (mm)	0.367 [0.267 to 0.407]	0.372 [0.339 to 0.432]
Trabecular separation (mm)	0.150 [0.124 to 0.173]	0.150 [0.121 to 0.169]
Trabecular inhomogeneity (mm)	0.060 [0.043 to 0.093]	0.048 [0.026 to 0.071]
Stiffness (kN/mm)	114.1 [99.3 to 140.0]	7.87 [5.45 to 9.00]
Ultimate failure load (kN)	5.99 [5.02 to 7.26]	0.79 [0.73 to 0.80]

Data are shown as median [interquartile range]

^a^Control data from previous study [10]

**Table 5:** High-resolution peripheral quantitative CT measurement parameters for the non-dominant distal radius for children with mild and severe osteogenesis imperfecta compared to controls

Parameter	Mild osteogenesis imperfecta	Severe osteogenesis imperfecta	Control^a^
Total volumetric bone mineral density (mgHA/cm^3^)	248.9 [213.8 to 264.3]	300.9 [232.5 to 369.3]	251.3 [234.4 to 285.0]
Cortical volumetric bone mineral density (mgHA/cm^3^)	688.3 [654.2 to 750.0]	658.9 [587.6 to 730.2]	699.6 [650.5 to 734.9]
Trabecular volumetric bone mineral density (mgHA/cm^3^)	138.3 [110.5 to 171.8]	211.4 [170.4 to 252.4]	166.6 [149.6 to 186.0]
Cortical thickness (mm)	0.43 [0.35 to 0.67]	0.49 [0.24 to 0.73]	0.50 [0.36 to 0.65]
Cortical porosity (no units)	0.457 [0.041 to 0.062]	0.057 [0.045 to 0.069]	0.347 [0.267 to 0.425]
Trabecular number (1/mm)	2.07 [1.94 to 2.08]	1.67 [1.23 to 2.10]	2.14 [1.97 to 2.30]
Trabecular thickness (mm)	0.056 [0.049 to 0.069]	0.108 [0.100 to 0.115]	0.064 [0.058 to 0.070]
Trabecular separation (mm)	0.427 [0.414 to 0.464]	0.536 [0.376 to 0.696]	0.407 [0.372 to 0.441]
Trabecular inhomogeneity (mm)	0.181 [0.169 to 0.244]	0.567 [0.417 to 0.716]	0.159 [0.135 to 0.174]
Stiffness (kN/mm)	36.28 [31.51 to 50.51]	44.11 [36.77 to 51.45]	55.64 [50.60 to 70.26]
Ultimate failure load (kN)	1.88 [1.67 to 2.58]	2.13 [1.81 to 2.44]	2.94 [2.62 to 3.62]

Data shown as median [interquartile range]

^a^Control data from previous study [10]

The study has limitations. The low consent rate might have been a result of the limited availability of the high-resolution peripheral quantitative CT scanner, which limited patient/parent choice as to when the scan could be performed, the location of the scanner not being at Sheffield Children’s Hospital and the fact that osteogenesis imperfecta patients at our institution are highly sought out for research. The low number and heterogeneity of recruits limits the strength of our conclusions. The use of two different dual-energy X-ray absorptiometry scanners might have systematically impacted the whole-body results despite the use of a conversion factor.

High-resolution peripheral quantitative CT itself has limitations that include the 3–5 h per patient required to produce a micro-finite element model and problems distinguishing the periosteal and endosteal perimeter of cortical bone in Type III patients, meaning the results for these parameters might be less reliable. This could be caused by trabecularisation of the cortex [13].

High-resolution peripheral quantitative CT analysis could be used in osteogenesis imperfecta to aid treatment planning and to monitor fracture risk and treatment response. The ankle might be more reliable than the wrist in children, with fewer repeated scans caused by motion artefact. Further study is required to fully understand the impact of bisphosphonates on skeletal microstructure and bone strength, and to ascertain the optimum site for imaging and how frequently scans should be performed, all in comparison to the current gold standard of dual-energy X-ray absorptiometry.

In conclusion, we have only conducted a small feasibility study, and although our impression is that it is more sensitive than dual-energy X-ray absorptiometry, a larger definitive study is required before recommendations can be made as to whether high-resolution peripheral quantitative CT should be used instead of or in addition to dual-energy X-ray absorptiometry. Future prospective studies might help to define changes in bone microarchitecture and strength following bisphosphonate therapy and to track individual aspects of treatment in children with osteogenesis imperfecta. Overall, we are encouraged that high-resolution peripheral quantitative CT scans are feasible in children (older than 8 years) with all forms of osteogenesis imperfecta.
